# Chitinolytic and Antagonistic Activity of *Streptomyces* Isolated from Fadama Soil against Phytopathogenic Fungi

**DOI:** 10.21315/tlsr2021.32.3.2

**Published:** 2021-09-30

**Authors:** Aminu Argungu Umar, Aminu Bandam Hussaini, Jibril Yahayya, Ibrahim Sani, Habiba Aminu

**Affiliations:** Department of Biochemistry, Kebbi State University of Science and Technology, P.M.B 1144, Aliero, Kebbi State, Nigeria

**Keywords:** Biocontrol, Chitinase, Enzyme Activity, Phytopathogenic Fungi, *Streptomyces*

## Abstract

Chitinases which degrade chitin have attracted attention as biological antifungal agents. The purpose of this study is to isolate *Streptomyces* from Fadama soil and assess its chitinolytic and antagonist potential against phytopathogenic fungi for application as biocontrol agent. *Streptomyces* were isolated from Fadama soil. The selected isolate CT02 exhibited chitinolytic characteristics. Chitinase production was performed under different temperatures, pH and varying incubation period. The highest chitinase production by CT02 isolate was observed after five days of cultivation. The highest chitinase activity was observed at 35°C and pH 7. The crude extracellular enzyme exhibited a specific activity of 4.20 U/μg whereas partially purified extracellular enzyme exhibited a specific activity of 6.19 U/μg with purification fold of 1.47. The selected isolate CT02 and its extracellular crude chitinase showed *in vitro* antifungal antagonist potential by inhibiting the growth of *Aspergillus niger* and *Aspergillus oryzae*. This indicates that *Streptomyces* derived chitinases are potential biocontrol agents against phytopathogenic fungi.

**Highlights**

- *Streptomyces* isolate CT02 isolated from Fadama soil exhibited chitinolytic activity.
- Crude and partially purified chitinase from *Streptomyces* isolate CT02 showed specific activity of 4.20 U/ug and 6.19 U/ug, respectively.
- *Streptomyces* isolate CT02 and its extracellular chitinase inhibited the growth of *Aspergillus niger* and *Aspergillus oryzae*.

## INTRODUCTION

The Gram-positive actinomycetes are aerobic bacteria that have high GC content in their genome and are good sources of secondary metabolites (Berdy 2005). *Streptomyces* in particular is the major producer of commercially important biomolecules (Aggarwal *et al*. 2016). *Streptomyces* are diverse, they have high rate of metabolic production and play an important role in organic matter recycling as well as being capable of degrading chitin and lignocelluloses (Khanna *et al*. 2011). Fadama is a Hausa word given to wetland soils that are low-lying swamp areas rich in fluvial deposits and exploitable aquifers (Gandi & Rajashekara 1989). These types of soils are found around rivers, swamps and floodplains (Ojanuga *et al*. 1996).

In Nigeria, farmers are more frequently facing the challenges of producing low quality plant products and yield losses due to the action of fungal phytopathogens. To date, over 10,000 phytopathogenic fungal species have been documented. Among them, *Aspergillus, Alternaria, Penicillium, Fusarium, Botrytis, Rhizopus Monilinia, Geotrichum, Mucor* and *Gloeosporium* are the most important genera (Barkai-Golan 2001). Chitin is a polymer of *N*-acetyl glucosamine and one of the most abundant biomaterials in nature. It is the common structural polysaccharide in exo-skeleton of insects and crustaceans and also as the major component of fungal cell wall (Shinya & Fukamizo 2017). Chitinase (E.C 3.2.1.14) is a glycosyl hydrolase (GH) that cleaves ß-1, 4 bonds between *N*-acetyl glucosamine residues in the chitin polymer. Chitinases are potential growth inhibitors of various pathogenic species that contain chitin as a component of their cell walls (Okay *et al*. 2013). More attention is now given to chitinases due to their potential as eco-friendly and safe biocontrol agents (Gupta *et al*. 1995; Cletus *et al*. 2013; Hammami *et al*. 2013; Awad *et al*. 2014; Nagpure *et al*. 2014).

Microbial enzymes have significant role in fundamental and industrial research. The aim of this study was to assess the chitinolytic and antifungal potential of Fadama soil *Streptomyces* in order to use them as biological control agents against phytopathogenic fungi. Isolation and characterisation of actinomycetes from unexplored habitats may present a vast opportunity to discover novel bioactive metabolites. Fadama land soils could be a good source of unique actinomycetes that can produce novel bioactive metabolites with potential applications as biocontrol agents against pathogenic microorganisms. There is no literature report on the existence of actinomycetes and their potential as biocontrol agents from the soil of Fadama lands reported in this study.

## MATERIALS AND METHODS

### Sample Collection

Chitin, *N*-acetyl glucosamine, 3, 5-dinitrosalicylic acid and nalidixic acid were obtained from Sigma-Aldrich. Fungal strains *Aspergillus niger* and *Aspergillus oryzae* were kindly provided by Dr. Aliyu Ibrahim Dabai of the Department of Microbiology, Usmanu Danfodio University, Sokoto, Nigeria. Soil samples were collected from Zauro (12.4318° N, 4.1956° E) and Yauri (10.8370° N, 4.7433° E) Fadama lands in Kebbi state Nigeria and transported in sterilised plastic bags to the laboratory. Soil samples were air dried overnight in foil trays and the following morning soils were placed in a drying oven at 60°C for 90 min then allowed to cool prior to use.

### Isolation of *Streptomyces*

About 1 g of soil was moistened with normal saline and mixed with 1 g of CaCO_3_ and incubated at 28°C for 30 min (Lechevalier & El-Nakeeb 1963). Sample was transferred into conical flask containing 100 mL normal saline and mixed by shaking and allowed to stand for 15 min. The suspended portion was serially diluted up to 10^−5^ (Kumar & Jadeja 2016). An aliquot of 0.1 mL of each dilution was inoculated on starch casein agar (SCA) plates containing nalidixic acid and incubated at 30°C for 14 days. Representative colonies were selected and streaked on a new SCA plates to obtain pure isolates of *Streptomyces*. A spore suspension was prepared using a sterile cotton swab moistened with glycerol, the spores were collected by gently rolling the moist sterile swab across the surface of the spore collection plate, one isolate at a time and liberated them by twirling the cotton swab into 0.75 mL of sterile 20% (v/v) glycerol. Screening for chitinolytic *Streptomyces* was carried out on a medium with colloidal chitin prepared as previously described (Murthy & Bleakley 2012). The cultures were incubated for 7 days at 28°C. After the incubation period, the appearance of clear halo zones around the colonies indicated the chitinolytic activity of the *Streptomyces*. The isolate that exhibited largest zone of chitin hydrolysis was selected for further physiological, biochemical and antifungal characterisations.

### Biochemical and Physiological Characterisation of the Selected Isolate

Starch hydrolysis, fermentation of carbohydrates, casein hydrolysis, catalase, methyl red and oxidase tests were conducted on the selected isolate. Also, growth of the selected isolate was assessed under varying conditions of temperatures (20°C–50°C), pH (3–9) and salt (1%–10% w/v KCl). The ability of the selected isolate to utilise carbohydrate was assayed using glucose, sucrose and fructose as carbon sources.

### Induction and Effects of Carbon Sources on the Production of Extracellular Chitinase

The cultivation of *Streptomyces* was performed using the production medium as described by Brzezinska *et al*. (2013), the medium contained (g/L) 0.1; yeast extract, 0.5; K_2_HPO_4_, 0.1; MgSO_4_.7H_2_O, 0.5; NaCl and 1% colloidal chitin (pH 7.5). A 100 mL of the media was inoculated with 1 mL of spore suspension and incubated for 7 days at 28°C with 120 rpm shaking. After incubation, the culture was centrifuged at 10,000 *g* for 10 min. The culture supernatant was filtered and used as crude extracellular chitinase in subsequent assay. The effects of different carbon sources were assessed; chitin, 1% colloidal chitin, starch, sucrose and glucose were individually added to basal culture medium containing (g/L) 0.1; yeast extract, 0.5; K_2_HPO_4_, 0.1; MgSO_4_.7H_2_O, 0.5; NaCl (pH 7.5). *Streptomyces* was inoculated separately into each of the cultivation medium with different carbon source and incubated in a shaking incubator (120 rpm) at 28°C for 7 days. One percent (1%) of colloidal chitin was found to be the most suitable carbon source for chitinase production by the isolated *Streptomyces* and was selected for assessing the optimisation of temperature (25°C, 30°C, 37°C, 40°C, 45°C and 50°C) and pH (5, 6, 7, 8, 9 and 10) of the medium. The culture supernatants were all separated from the cell debris by centrifugation at 12,000 *g* for 10 min. The supernatants were filtered and assayed for extracellular chitinase activity expressed as U/mL. Protein concentration was determined following the method of Bradford (Bradford 1976). The culture filtrate was fractionated with 50%–80% ammonium sulphate saturation. The precipitate of 70%–80% was centrifuged at 12,000 *g* for 10 min. The precipitate was dialysed 3x in deionised water and lyophilised to powder and finally, the powder was re-suspended in 0.02M Tris buffer (pH 7.5).

### Chitinase Activity Assay

Chitinase activity was determined following the method described by Liu *et al*. (2015) with minor modification. Briefly, the reaction mixture of 1 mL extracellular enzyme solution and 1 mL of 1% colloidal chitin was set up in 10 mM sodium citrate buffer at pH 7.0 and incubated at 37°C for 1 h. The reaction was terminated with the addition of 0.2 mL 3,5-dinitrosalicylic acid (DNS) followed by heating in boiling water for 10 min. Samples were cooled rapidly by immersing the reaction tubes into ice water then centrifuged at 10,000 *g* for 10 min. The absorbance of the supernatant containing released *N*-acetyl-d-glucosamine (GlcNAc) was measured at 540 nm using a Multiskan EX microplate reader (Thermo Scientific, USA). Appropriate blanks were incorporated to subtract possible unwanted absorbance due to factors other than specific hydrolysis of GlcNAc. The amount of released reducing sugars *N*-acetyl-d-glucosamine (GlcNAc) was determined by the DNS method (Miller 1959). One unit of chitinase activity represents the amount of enzyme that liberated 1 μmol of reducing sugar per minute.

### Partial Purification of Chitinase with Ammonium Sulphate

The culture medium was precipitated with 80% saturation of ammonium sulphate. The precipitate was obtained by centrifugation at 15,000 *g* for 20 min and dissolved in 50 mM sodium phosphate buffer (pH 7.0) and immediately dialysed against the same buffer.

### Biochemical Characterisation of Chitinase

The optimum temperature for chitinase activity was determined by setting up reactions at different temperatures (20°C–60°C) at pH 7. Thermal stability of the chitinase enzyme was assessed by incubating the enzyme separately at 40°C, 45°C, 50°C, 55°C, 60°C, 65°C and 70°C for 3 h followed by activity assay. Similarly, the optimum pH for chitinase activity was assessed by setting the enzyme reactions at various pH ranges (4–9). The pH stability of chitinase was measured at different pH values by incubating the chitinase in buffers of different pH for 3 h at 35°C prior to setting up reactions to assay the activity.

### Antifungal Activity Assays

Antifungal performance of chitinase was tested by hyphal extension-inhibition assay as described by Zhang *et al*. (2013). Each of the fungal strain (*Aspergillus niger* and *Aspergillus oryzae*) was separately streaked on PDA plate and incubated at 28°C for 3 days. Each grown fungus from individual PDA plate was collected using a sterile toothpick and transferred to a new PDA plate and incubated till fungal growth was observed. On each PDA plates with fungal growth, 50 μL of spore suspension was added at three different positions. On other separate PDA plates with fungal growth, three wells of about 0.8 cm depth were perforated and added 20 μL of crude and partially purified chitinase into the wells. The plates were incubated at 28°C for 5 days.

## RESULTS

### Isolation and Screening of *Streptomyces*

**Figure 1:**
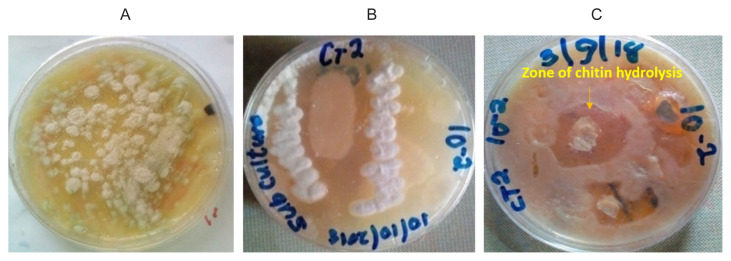
(A) *Streptomyces* CT02 isolates; (B) Sub-cultured *Streptomyces* CT02 isolate; and (C) Chitinolytic screening plate showing zone of chitin hydrolysis by CT02.

A total of twelve strains of chitinolytic *Streptomyces* were isolated, five from Zauro and seven from Yauri Fadama soil samples (Table 1). The representative isolates are presented in Fig. 1(A). Isolates were further purified by subculture [Fig. 1(B)]. *Streptomyces* were screened for chitinolytic activity and the strain that exhibited the highest chitinase production was identified based on the clear zone of hydrolysis of chitin on colloidal chitin plates [Fig. 1(C)], this strain was selected for further studies.

### Biochemical and Physiological Characterisation of the Selected Isolate

The isolated strain was subjected to biochemical and physiological characterisation. The selected isolate CT02 was non-motile Gram positive, chalky, earthy odour with aerial and substrate mycelium formation. The growth rate was fair, and the colony colour was white. The isolate hydrolyse starch and casein and exhibited catalase activity but oxidase was not produced. The isolate was able to utilise glucose, sucrose and fructose as carbon sources but was negative to methyl red test. The isolate can grow at temperature and pH ranges of 20°C–50°C and 5–9, respectively. It shows tolerance of up to 8% KCl (Table 2).

### Induction and Effects of Carbon Sources on Extracellular Chitinase Production

Chitinases are inducible class of enzymes. The synthesis and activities of chitinases are stimulated by the availability of appropriate substrate. Efficient production of chitinase was observed from the isolated *Streptomyces* that was cultivated on mediums containing chitin, 1% colloidal chitin, and starch as carbon and nitrogen sources. The chitinolytic activity was not observed in the medium containing sucrose and glucose as carbon and nitrogen sources. This shows that this enzyme is inducible, and that chitin, colloidal chitin, and starch are more suitable inducers of chitinase production. This could be attributed to the presence of 1, 4-linked monosaccharide units in chitin, colloidal chitin, and starch which is not found in sucrose and glucose.

### Biochemical Characterisation of Chitinase

**Figure 2:**
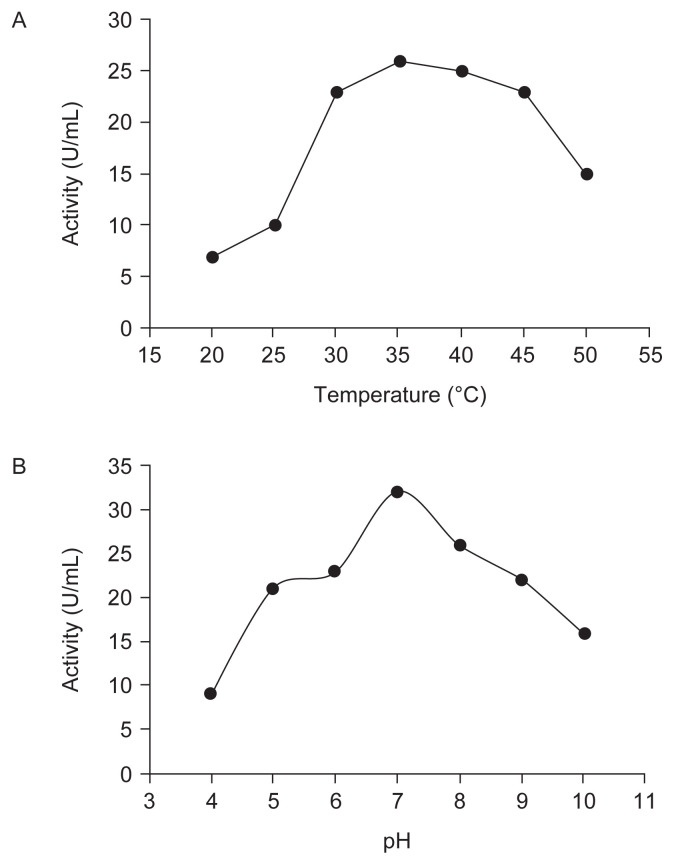
(A) Effect of temperature on chitinase activity of *Streptomyces* isolate CT02; (B) Effect of pH on chitinase activity of *Streptomyces* isolate CT02.

The extracellular chitinase produced by the selected isolate (CT02) was purified by ammonium sulphate precipitation. The protein content and enzyme activity were determined. The crude enzyme exhibited a specific activity of 4.20 U/μg but ammonium sulphate precipitated chitinase exhibited a specific activity of 6.19 U/μg with purification fold of 1.47. Chitinase of the selected isolate CT02 showed activity at temperature ranges 20°C–50°C, although it exhibited declined activity at 50°C but showed optimum temperature for the activity to be 35°C [Fig. 2(A)]. To determine the thermal stability of the enzyme, the partially purified chitinase was incubated at different temperatures (40°C, 45°C, 50°C, 55°C, 60°C, 65°C and 70°C) for 3 h prior to activity assay. Our results indicated that much of the chitinase activity was maintained after incubation at 50°C. This is consistent with the activity observed between temperatures 20°C–50°C in Fig. 2(A). However, the activity was lost after the enzyme was incubated at 60°C and 70°C prior to activity assay, indicating that the chitinase enzyme is not stable at these temperatures. Next, the optimum pH for chitinase activity was assessed by setting the enzyme reactions at various pH ranges (4–9). Similarly, the pH stability of the chitinase was measured at different pH values by incubating the chitinase in buffers of different pH for 3 h at 35°C prior to setting up reactions to assay the activity. The activity of CT02-produced chitinase was considerably observed in the pH range 4–10 with optimum activity exhibited at pH 7 [Fig. 2(B)] and it shows considerable stability at between pH 6–8 when incubated for 3 h at these pH ranges prior to reactions. Temperature (°C)

### Antifungal Activity Assays

**Figure 3:**
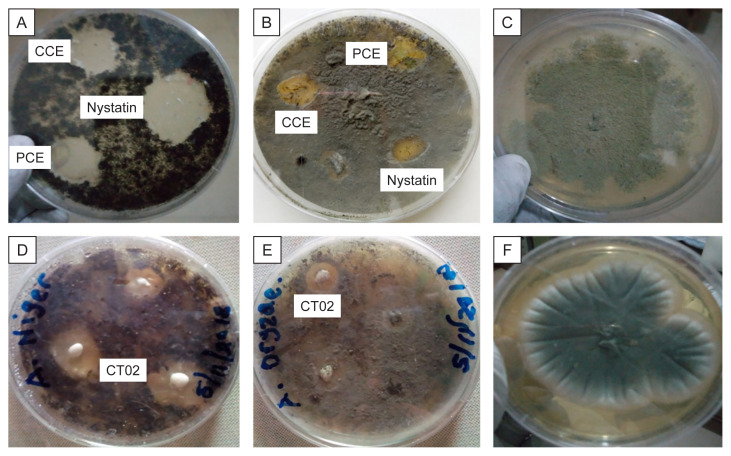
Antifungal screening.: (A) Antifungal screening for crude chitinase enzyme (CCE), purified chitinase enzyme (PCE) and standard antifungal drug (Nystatin) against *Aspergillus Niger*; (B) Antifungal screening for crude chitinase enzyme (CCE), purified chitinase enzyme (PCE) and standard antifungal drug (Nystatin) against *Aspergillus Oryzae*; (D) Antagonist activity of CT02 spore suspension against *Aspergillus Niger;* (E) Antagonist activity of CT02 spore suspension against *Aspergillus Oryzae;* (C and F) Controls, using basal medium (without chitinase or spore) against the growth of *Aspergillus Niger* and *Aspergillus Oryzae*, respectively.

The spore suspension, crude and partially purified chitinase of CT02 exhibited antagonist activity against the growth of the tested fungal strains (Table 3). The crude and partially purified chitinase showed high degree of inhibition against *Aspergillus niger* with almost the same degree of inhibition, but Nystatin (Sigma-Aldrich) which is a standard antifungal drug exhibited higher degree of inhibition on *Aspergillus niger* [Fig. 3(A)]. On the other hand, crude and partially purified chitinase of CT02 exhibited moderate inhibitory effect on the growth of *Aspergillus oryzae*. The spore suspension of CT02 showed a clear zone of inhibition against *Aspergillus niger* and moderate inhibition on the growth of *Aspergillus oryzae* [Figs. 3(D) and 3(E), respectively]. The zone of inhibitions was measured (Table 3).

## DISCUSSION

Fadama soils are biologically enriched, fertile and favourable for the flourishment of plants and microbes due to the abundance of water from rivers, this enhances the growth of plants and microbes which in turn result to the development of fertile soils (Jamala *et al*. 2012). The biotope studied in this research includes a sandy loam and clay loam soils from Zauro and Yauri localities, Kebbi Sate, Nigeria. Isolation of chitinase producing actinobacteria from these ecosystems is interesting in order to identify bacteria with a unique metabolism that arise as a result of adaptation to changes in the condition of temperatures in those climates. In these climates, temperature can rise up to 45°C and above during the summer and drop to as low as 15°C during harmattan period. These changes in temperature conditions can give rise to adaptation by microorganisms which can also lead to evolving unique traits of microbes. The CT02 isolate characterised in this study, has shown adaption to high and low temperatures with ability to grow at 20°C and 50°C. It has been reported that strains of actinobacteria such as *Streptomyces, Acidothermus cellulolyticus* and *Micromonospora* that are able to degrade chitin exhibited ability to grow at above 45°C (Mohagheghi *et al*. 1986; Goodfellow *et al*. 1987; Singh *et al*. 2014; Meriem *et al*. 2019).

The data obtained in present study indicated that CT02 produced chitinase enzyme and that the induction of chitinase synthesis depends on factors such as medium composition and source of chitin. Previous studies have shown that optimum chitinase production by *Streptomyces* was achieved using medium containing colloidal chitin (Narayana & Vijayalakshmi 2009; Han *et al*. 2009; Meriem & Mahmoud 2017; Meriem *et al*. 2019; Brzezinska *et al*. 2019). Like all enzymes, temperature and pH affects the activity of chitinases. We show here that the chitinase produced by the isolate CT02 exhibited activity at temperature ranges 25°C–40°C with optimum activity observed at 35°C. The enzyme showed considerable stability at temperature of up to 50°C because much of the chitinase activity was maintained after the enzyme was pre-incubated at 50°C prior to the start of the reaction. However, activity was lost when the enzyme was incubated at 60°C and 70°C prior to the reaction, indicating that the chitinase enzyme is not thermostable at these temperatures. Similarly, our result shows that the activity of CT02-derived chitinase was considerable between pH 5–9 with optimum activity observed at pH 7, it also shows stability at pH 6–8 when incubated at these pH range prior to reactions start. Various research groups have reported that the reaction temperature and pH of *Streptomyces* derived chitinases are in the ranges of 30°C–60°C and 2–12.5, respectively (Rabeeth *et al*. 2011; Xiayun *et al*. 2012; Prakash *et al*. 2013; Pradeep *et al*. 2014; Karthik *et al*. 2015; Rashad *et al*. 2017). However, Brzezinska *et al*. (2019) showed that chitinase from *S. luridiscabiei* U05 was not stable at 50°C. The differences in optimum temperature and thermostability of *Streptomyces* derived chitinases recorded by different studies may be due to the different sources from which the *Streptomyces* was isolated. It is likely that some strains of *Streptomyces* have evolved to adapt to extreme or low temperatures according to their habitat.

Although, *Aspergillus* species are not regarded as the major phytopathogenic fungi, they causes various disorders in variety of plants (Kozakiewicz 1989). The most common species are *A. flavus, A. niger*, *A. ochraceus, A. parasiticus*, *A. alliaceus*, and *A. carbonarius*. They can cause contamination during pre-harvest, harvest, processing or handling of agricultural products (Kozakiewicz 1989). Various studies have reported the antifungal effect of chitinases derived from bacteria (Nagpure & Gupta 2013; Karthik *et al*. 2015; Nguyen *et al*. 2015; Rostami *et al*. 2017). The antagonistic performance of *Streptomyces* species against pathogenic fungi is associated to the production of antifungal metabolites. Chitinases and other metabolites of *Streptomyces* may synergistically enhance the antagonist activity against plant fungal pathogens. Several studies have previously reported the antifungal activity of chitinolytic *Streptomyces* species against different plant pathogenic fungi (Rabeeth *et al*. 2011; Pradeep *et al*. 2014; Yandigeri *et al*. 2015; Rashad *et al*. 2017; Brzezinska *et al*. 2019; Boukaew *et al*. 2020; Danial *et al*. 2020). This suggests the potentials of *Streptomyces* species as biocontrol agents. Our study revealed that antifungal chitinase was produced by CT02. Chitinases exhibited promising potentials in pest control, however, these enzymes are yet to be fully exploited commercially due to low production yield and high cost of production (Muffler *et al*. 2015). In order to harness the full benefit in the application of the *Streptomyces*-derived chitinase, the purified enzyme need to be immobilised appropriately to preserve its activity. To date, our study could be the first ever report on the isolation and characterisation of chitinolytic and antifungal *Streptomyces* from Fadama soils.

## CONCLUSION

Results of the present study showed that the partially purified chitinase and culture filtrate of the isolate CT02 exhibited antagonist activity against *Aspergillus niger* and *Aspergillus oryzae*. Our study suggests that *Streptomyces* isolate CT02 from Fadama ecosystem present an opportunity that can be harnessed to identify and isolate a novel actinobacteria for potential production of high-grade novel bioactive compounds for applications as biocontrol agents. Further research is highly recommended to isolate, identify and characterise the actinobacteria-derived bioactive compound.

## Figures and Tables

**Table 1:** Screening of chitinolytic *Streptomyces*.

Symbol	Source	Zone of chitin hydrolysis (mm)
ST01	Zauro Fadama	3
ST02		1
ST03		4
ST04		2
ST05		1
CT01	Yauri Fadama	2
CT02		6
CT03		1
CT04		3
CT05		2
CT06		1
CT07		3

**Table 2:** Biochemical and physiological characterisation of the selected isolate.

Test	Result
Gram staining	+
Starch hydrolysis	+
Casein hydrolysis	+
Catalase	+
Methyl red	−
Oxidase	−
Carbon utilisation	
Glucose	+
Sucrose	+
Fructose	+
Temperature tolerance	
Growth at 20°C–50°C	+
pH tolerance	
Growth at pH 3–4	−
Growth at pH 5–9	+
KCl tolerance	
Salt tolerance at 1%–8%	+
Salt tolerance at 9%–10%	−

**Table 3:** Growth Inhibition of the tested fungal plant pathogens.

Fungal strain	Growth inhibition (mm)		

	Crude chitinase enzyme	Partially purified chitinase	Spore suspension
Aspergillus niger	13.0 ± 1.5	11.5 ± 1.3	11.0 ± 1.5
Aspergillus oryzae	5.0 ± 1.2	5.0 ± 1.5	6.0 ± 1.2

*Note*: Antifungal activity was categorised as; − = no inhibition, ±<2 mm = weak inhibition, +2–10 mm = moderate inhibition, ++>10 mm = strong inhibition. ± standard deviation (n = 3) (Adapted from Ghasemi *et al*. 2011).
